# Supplementary material for: Antioxidant and Anti-Inflammatory Activity of Coffee Brew Evaluated after Simulated Gastrointestinal Digestion
Source: Nutrients. 2021 Dec 5;13(12):4368. doi: 10.3390/nu13124368 (PMC8705407; doi:10.3390/nu13124368)
Supplement: Supplementary file 1 [file nutrients-13-04368-s001.zip › nutrients-1463152-supplementary.pdf]

Supplementary materials:

Table S1. Stock solutions composition

Salt solution	Stock concentration (mol/L)	SSF (pH 7)		SGF (pH 3)		SIF (pH 7)	
		mL of Stock added to prepare 0.4 L (mL)	Final salt concentration in sample (mmol/L)	mL of Stock added to prepare 0.4 L (mL)	Final salt conc. in sample (mmol/L)	mL of Stock added to prepare 0.4 L (mL)	Final salt conc. in sample (mmol/L)
KCl	0.5	15.1	15.1	6.9	6.9	6.8	6.8
KH_2PO_4	0.5	3.7	1.35	0.9	0.9	0.8	0.8
$NaHCO_3$	1	6.8	13.68	12.5	25	42.5	85
NaCl	2	-	-	11.8	47.2	9.6	38.4
$MgCl_2(H_2O)_6$	0.15	0.5	0.15	0.4	0.12	1.1	0.33
$NH_4(CO_3)_2$	0.5	0.06	0.06	0.5	0.5	-	-
$CaCl_2(H_2O)_2$	0.3	-	1.5	-	0.15	-	0.6

Table S2. TPC and antioxidant capacity evaluated by FRAP, DPPH and ABTS of the Blank control.

Samples	FRAP		DPPH		ABTS		TPC	
	**mmol TE/100g**	**±SD**	**mmol TE/100g**	**±SD**	**mmol TE/100g**	**±SD**	**mg GAE/g**	**±SD**
Blank control	1.07	0.01	0.39	0.01	1.02	0.01	0.07	0.00
